# Impact of Morphology and Composition of Graphene Aerosol–Gel Particles in Thin Films on Ultrafast Carrier Dynamics Studied via Transient Absorption Spectroscopy

**DOI:** 10.1021/acs.jpcc.6c02864

**Published:** 2026-06-05

**Authors:** Alexander J. Auty, Negar Mansouriboroujeni, Thiba Nagaraja, Dimitri Chekulaev, Natalia Martsinovich, Suprem R. Das, Adrien A. P. Chauvet

**Affiliations:** † School of Mathematics and Physical Sciences, 7315 University of Sheffield, Sheffield S3 7HF, U.K.; ‡ Department of Industrial and Manufacturing Systems Engineering, 5308 Kansas State University, Manhattan, Kansas 66506, United States; § Department of Electrical and Computer Engineering, 5308 Kansas State University, Manhattan, Kansas 66506, United States

## Abstract

Carrier dynamics of a series of printed thin films made of graphene aerosol–gel inks with varying oxygen (with respect to carbon in the lattice) content were investigated via ultrafast pump–probe spectroscopy. This study builds upon previously reported work which compares printed graphene aerosol–gel films to pristine graphene films with atomically flat flake physical structure. The present study thus further investigates the tunability of graphene aerosol–gel films and presents a systematic extension using oxygen as the tuning element. We report on the transient change in the transmission (Δ*T*/*T*) of printed inks with various oxygen content spanning the visible to near-IR spectral range (3–0.8 eV). The transient electronic behavior is correlated with composition, shape and morphology of ink particles, and sp^2^ carbon quality. We demonstrate that higher oxygen content directly correlates with increased concentration of long-lived trapped electronic states. Based on density functional theory calculations, these trapped states may be attributed to the presence of substitutional oxygen.

## Graphical abstract

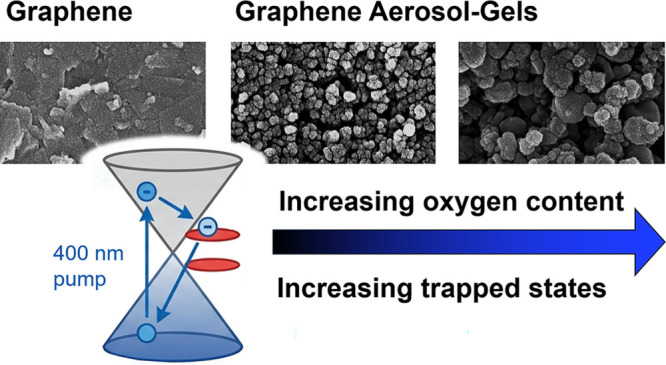

## 1 Introduction

Ultrafast pump–probe studies of charge carrier dynamics in electronic material systems have been transformative in exploring electronic properties of materials, the tunability of their physical and molecular structure, and the relation between their atomic and electronic structure, leading to unprecedented opportunities both for fundamental science and technological applications.^1–6^ This is especially true for low dimensional structures, like graphene, which can demonstrate quantum confinement perpendicular to their basal plane, crystal symmetry breaking at the nanoscale, and larger surface area for interaction with their environment compared to their bulk counterparts. The electronic properties of these low-dimensional materials can be further tuned by engineering of their ideal 2D structure through, for example, atomic defects, inclusion of other atoms in host crystal lattice, and nanoscale deformations; all of which can fundamentally alter the charge dynamics in these atomic crystals.^7,8^ Graphene, owing to its relatively high optical phonon energy of ∼200 meV compared to traditional semiconductors and metals, is of particular interest in optical pump–probe studies in elucidating the carrier dynamics and relaxation pathway of photoexcited electrons, as summarized in Figure 1.

**Figure 1:**
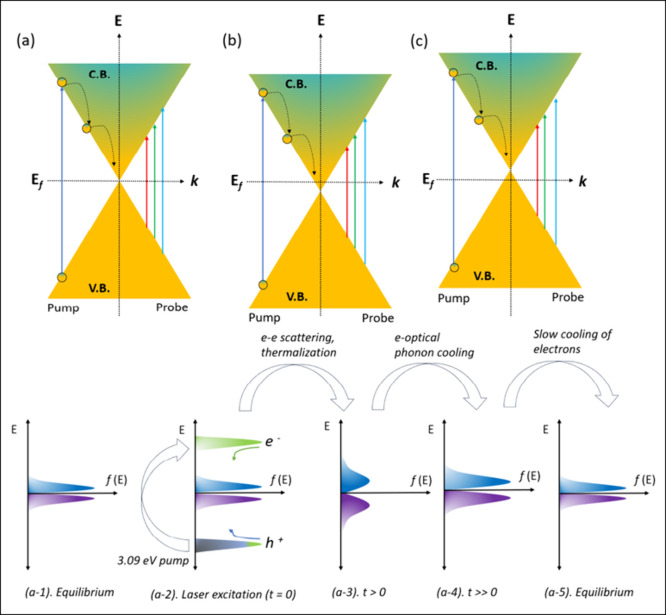
(upper panel) Linear energy-momentum band structure of (a) intrinsic graphene, (b) n-type graphene, and (c) p-type graphene. Colored arrows depict the interaction of the pump, and the probe laser pulses with the above electronic structure of graphene. (lower panel) Evolution of the carrier (electron and hole) population and cooling leading to equilibrium as a function of pump–probe delay, *t*, energy, *E*, and Fermi–Dirac distribution function, *f* (*E*).

While previous studies have explored the ultrafast carrier dynamics of graphene and graphene oxide materials,^9–13^ a comprehensive understanding of how the variation in the oxygen content in sp^2^ carbon lattice structure of graphene aerosol gel films remains elusive. The details of an eco-friendly manufacturing processes of aerosol gel materials (in powder form) and aerosol gel inks have been previously reported, and a comparison of ultrafast pump–probe spectroscopic studies between graphene ink with atomically thin flakes and an aerosol gel ink has been recently reported by the authors.^1,2,14,15^ The present study thus corresponds to an extension of these prior works, which addresses the gap in knowledge by systematically investigating a series of graphene aerosol–gel ink based printed films with controlled oxygen content. Although, as will be shown subsequently, varying oxygen content directly influences the films’ morphology. The discussed modulations of electronic behavior are correlated to oxygen content more than to differing morphologies based on previous studies.^1^ Altogether, this work provides a comprehensive correlation between the films’ oxygen content and the resulting ultrafast carrier relaxation pathways. These results are benchmarked against similar printed films but made of graphene ink with atomically flat graphene flakes manufactured by a liquid phase exfoliation method.

## 2 Experimental Methods

### 2.1 Preparation of Exfoliated Graphene and Graphene Aerosol–gel Films

Synthesis of graphene flakes using liquid phase exfoliation: 1 g of ethyl cellulose (EC, 4 cP, Sigma-Aldrich) was dissolved in 200 mL of ethanol via bath sonication for 30 min. Subsequently, 10 g of commercial graphite (99% carbon basis, ∼325 mesh particle size (50–70%), Sigma-Aldrich) was added to the EC solution. The resulting suspension was exfoliated using probe sonication (Q500 Sonicator, 500 W, 20 kHz, QSonica) for 6 h in pulse mode (5 s on and 5 s off) at 100% amplitude, with the suspension maintained in an ice bath. The exfoliated graphene/EC suspension was centrifuged (Microfuge 16, Beckman Coulter) at 11641 rpm for 15 min to separate unexfoliated graphite from graphene flakes. The supernatant containing graphene was carefully collected. A sodium chloride (NaCl, Thermo Fisher Scientific) solution was then prepared at a concentration of 0.04 g/mL in a volume twice that of the collected supernatant. The graphene/EC supernatant was slowly added to the NaCl solution under magnetic stirring to induce flocculation of the graphene flakes. The flocculated graphene/EC material was vacuum filtered using a 0.45 μm mixed cellulose esters (MCE) hydrophilic filter membrane (Sigma-Aldrich), with 500 mL of deionized water used to rinse away residual salt. The collected graphene/EC powder was then dried on a hot plate at 50 °C overnight. To further eliminate large flakes, the dried graphene/EC powder was first redispersed in 50 mL ethanol and bath sonicated for homogeneous suspension. This dispersion was passed through a 5 μm syringe filter, followed by another round of flocculation, vacuum filtration, and drying as previously described.

Stock graphene/EC ink of 100 mg/mL was prepared from obtained graphene/EC powder using cyclohexanone (99%, Sigma-Aldrich) and terpineol (mixture of isomers, anhydrous, Sigma-Aldrich) mixture (ratio of 85:15 v/v) as a solvent, via bath sonication. Then 20 μL of above stock ink was added to 100 μL of cyclohexanone and terpineol mixture to attain further diluted ink.

Synthesis of graphene aerosol–gel flakes using liquid phase exfoliation: The graphene aerosol–gel/EC powder (henceforward, AG/EC) was synthesized using a slight variation of graphene/EC synthesis protocol described above: the source material was produced from a gas phase acetylene and oxygen codetonation process instead of commercially purchased graphite, as described by Wright et al.^14^ The codetonation used a range of different molar ratios of the precursors, particularly, oxygen to acetylene ratio, resulting in graphene aerosol–gel based powders with varied morphological and chemical compositions.^14^ The molar ratio, before detonation, is herein denoted as (O_2_/C_2_H_2_) = O/C. The different O/C ratios investigated in this work are 0.3, 0.4, 0.5, and 0.75. Briefly, for the ink synthesis, 100 mg of EC was first dissolved in 50 mL of ethanol via bath sonication. 250 mg of graphene aerosol gel was added to the EC solution and was subjected to probe sonication for 3 h at 50% digital amplitude with 5 s pulses on and off mode in an ice bath. The resulting AG/EC suspension was filtered through a 5 μm syringe filter to remove large AG/EC flakes, followed by flocculation using a magnetic stirrer for 5 min with an aqueous NaCl solution (0.04 g/mL), maintaining a 1:2 volume ratio of AG/EC suspension and the NaCl solution. The flocculated AG/EC flakes were then vacuum filtered using a 0.45 μm mixed MCE hydrophilic filter membrane along with deionized water. The stock AG/EC ink with a concentration of 106 mg/mL was prepared by adding the dried powder to cyclohexanone and terpineol mixture followed by bath sonication. Diluted AG/EC ink was prepared by combining 20 μL of stock ink in 200 μL of cyclohexanone and terpineol mixture. The steps described above were applied consistently to all the different O/C ratios (0.3, 0.4, 0.5, and 0.75). The resulting AG samples are labeled AG 0.3, AG 0.4, AG 0.5, and AG 0.75.

Graphene (G) and graphene aerosol gel film preparation: The G/EC and AG/EC inks obtained by the above-described methods were then printed on precleaned quartz substrates (1 × 1 in. × 0.5 mm thick, SPI Supplies) using a Microplotter system (Microplotter II, Sonoplot) with a glass micropipette of 40 μm nozzle diameter. Each film corresponds to 3 printing passes of 5 mm by 5 mm square patterns. The printed patterns were annealed in a tube furnace with argon/hydrogen atmosphere for 2 h at 350 °C to remove the EC polymer.

### 2.2 Structural Characterization of Printed Patterns

Hitachi SU8230 field emission scanning electron microscopy (FESEM) was used at an accelerating voltage of 5 kV on the 2 nm iridium-coated printed patterns to study the morphology of printed patterns. A Renishaw InVia Raman microscope with an excitation wavelength of 532 nm was used to analyze the structural fingerprints of the printed graphene (aerosol gel ink material) patterns.

### 2.3 UV–Vis Spectroscopy

UV–vis absorption measurements were taken using an Agilent Cary 60 UV–vis spectrometer in the range between 200 and 800 nm.

### 2.4 Pump–Probe Spectroscopy

UV–vis pump–probe spectroscopy experiments were conducted at the Lord Porter Ultrafast Laser Laboratory (ULS), The University of Sheffield, using a Helios system (HE-VIS-NIR-3200) provided by Ultrafast Systems. The experiments utilized a Ti:sapphire regenerative amplifier (Spitfire ACE PA-40, Spectra-Physics) for producing 800 nm pulses (40 fs fwhm, 10 kHz, 1.2 mJ). The 400 nm pump pulses (2.5 kHz, 0.2 μJ) were generated through frequency doubling of the amplifier’s ∼800 nm output. The pump was focused onto the sample film to a spot size of approximately 150 μm in diameter. The white light probe continuum (440–700 nm) was generated using a sapphire crystal and a portion of the amplifier’s output. The intensity of the probe light transmitted through the sample was measured using a CMOS camera, with a resolution of 1.5 nm. The NIR probe continuum (850–1600 nm) was generated using a sapphire crystal. Prior to the generation of the probe continuum, the 800 nm pulses were passed through a computer controlled optical delay line (DDS300, Thorlabs), which provides up to 8 ns of pump–probe delay. The instrument response function is approximately 100 fs, for both the UV–vis and near-IR measurements, based on the temporal duration of the coherent artifact signal from the quartz substrate. All steady state and time-resolved spectroscopic data was processed using OriginPro. Preprocessing of the pump–probe data was performed using SurfaceXplorer, the software package provided by Ultrafast Systems. Kinetic traces from the pump–probe data were fit in OriginPro using a sum of decaying exponential functions convoluted with a Gaussian function. The Gaussian function was used to model the instrument response growth of the transient signal. The function can be found in the Supporting Information (SI).

### 2.5 Computational Method

Density functional theory (DFT) calculations were carried out with SIESTA software, using the vdW-DF2 functional.^16,17^ The calculations used norm-conserving pseudopotentials optimized using the GGA-PBE approximation and double-ζ polarized basis sets optimized for these pseudopotentials, obtained from Simune Atomistic database.^18^ The simulation systems consisted of 32 atoms in a rectangular unit cell periodic in two dimensions, with a vacuum thickness of 30 Å in the vertical dimension. Geometry optimization was carried out using the conjugate gradient algorithm, with a maximum atomic displacement of 0.1 Å per step, with the convergence criterion being the maximum atomic force being below a 0.01 eV/Å threshold. All atoms were allowed to be optimized, while the lattice parameters of the simulation cell were fixed at their ideal values optimized in pristine graphene (8.838 Å × 9.520 Å for the 32-atom rectangular cell). A Monkhorst–Pack *k*-point grid of 24 × 24 × 1 was used in all calculations. Band structures were calculated with 40 points along each segment of the line connecting special points (Γ-P-X-S-Y-Γ) and analyzed using the gnubands tool, which is a part of the SIESTA toolkit.

## 3 Results and Discussion

The UV–vis transmission spectra of the printed graphene (G) and AG films (AG 0.3–0.75) are given in Figure 2. In line with previous reports, G displays broad absorption in the 200–800 nm region, with a minimum in the transmission spectrum at 267 nm.^3,6,19,20^ Common to all AG films is a broadening of the transmission spectrum relative to G, with 0.75 exhibiting the most drastic changes. AG 0.3–0.5 exhibit transmission spectra minima at 253 nm, slightly blue-shifted relative to G and very similar to the previously reported AG film.^1^

**Figure 2:**
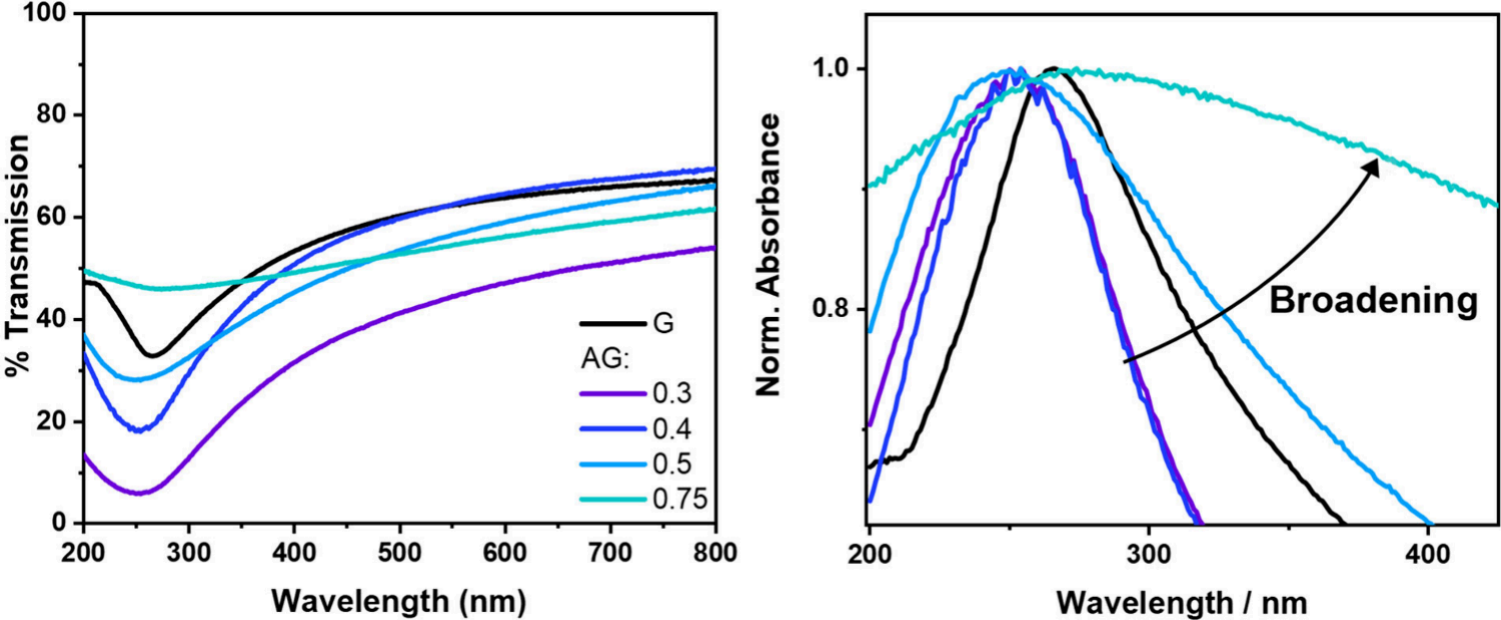
(left panel) UV–vis absorption spectra of inkjet print aerosol gel graphene films, with varying O/C ratio, and an inkjet printed pure graphene film. (right panel} Normalized graph of the UV–vis absorption spectra.

AG 0.75 has a significantly broader spectrum, resulting in less prominent transmission minima, which nearly coincide with that of G. The broadening of the spectra may be partly due to changes in the particle size. Indeed, the particle size of the different graphene samples increases as a function of the O/C ratio, as shown in Figure 3, which may either be the result of a modified electronic structure, as previously discussed,^1^ without excluding the possibility of direct scattering of the probing light by the particles.

**Figure 3:**
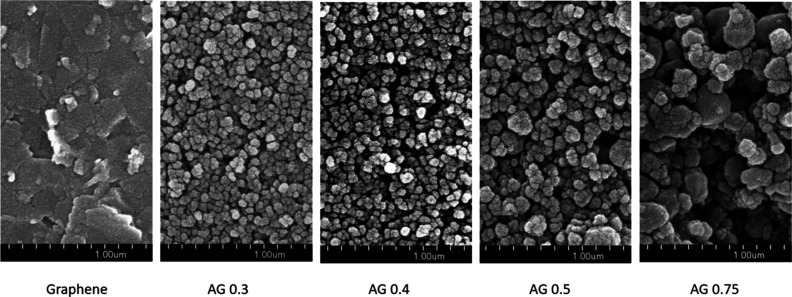
SEM images of graphene printed film and AG printed film.

### 3.1 Scanning Electron Microscopy (SEM)

The SEM images (Figure 3) of printed graphene thin films consisting of G or AG ink particles with different O/C ratio show that, while G primarily forms a network of interconnected atomically flat flakes, the AG films consist of particles with spherical shapes that are well packed and physically interconnected, with average particle sizes increasing as the O/C ratio increases.^15^ As the oxygen content is increased, there is a greater opportunity for the oxygen to react with the carbon atoms in the graphene flakes, leading to the observed increase in particle size. The shape of the particles arises from the ink manufacturing process employed, which used an ultrasonication based shockwave propagation and energy transfer to constituent precursor materials.^21,22^ The average particle sizes for the AG films shown in Figure 2 range from 0.1 μm (for O/C 0.3) to 0.4 μm (for O/C 0.75).

### 3.2 Raman Spectroscopy

Raman spectroscopy is widely used as one of the important fingerprint techniques to identify the quality of graphene materials.^23,24^ The G-peak is related to the vibrational mode of sp^2^-bonded carbon atoms in the graphene lattice and is located at around 1600–1700 cm^–1^, as shown in Figure 4. For monolayer graphene the G-peak is observed at ∼1580 cm^–1^ and is symmetrical in shape. The G-peak position and symmetry are often used as a reference to measure changes in the sp^2^ carbon structure and quality of graphene-based materials, or to quantify the number of graphene layers (typically for very few layers).^24^ The G-peak is associated with the in-plane stretching vibration of sp^2^ hybridized carbon atoms in the graphene lattice.^25^ Therefore, any deformation, such as mechanical bending-induced strain, stacking deformation-induced strain, and chemical compositional changes (such as oxygen or any other elemental presence in sp^2^ carbon lattice) can alter the G-peak position and line shape.^26^

**Figure 4:**
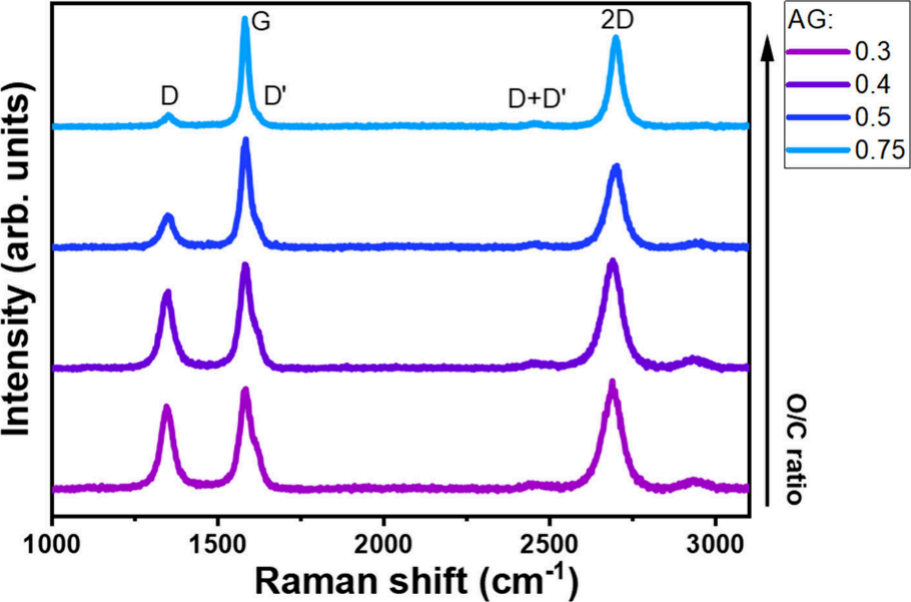
Raman spectroscopic measurement of the AG samples with O/C ratio of 0.3, 0.4, 0.5, and 0.75 after annealing.

The D-peak is related to the presence of defects or disorder in the graphene lattice, including the edges of flakes, and is located at ∼1350 cm^–1^.^27^ It arises due to the breathing mode of the six-membered rings in the graphene structure, which is activated by the introduction of defects or disorder.^24^ The shift of the D-peak to higher or lower frequencies relative to its expected position can provide information about the degree of disorder or strain in the graphene lattice.^28^ Together, the relative amplitudes of D to G-peaks give an appreciation of the degree of disorder in the material.^29^ Similar to the D-peak, the position and shape of the 2D-peak in the Raman spectrum can also provide information about the number of layers, the presence of strain or doping, and the electronic band structure of graphene.^24^ The 2D-peak is located at ∼2700 cm^–1^ for monolayer graphene, and its position shifts to lower frequencies for bilayer and few-layer graphene. This shift is due to the interaction between adjacent layers and the reduction of the interlayer coupling in thicker graphene samples.^30^ Overall, the Raman characterization of our AG films shows the presence of G, 2D, and D peaks with the least defect density and more graphene-like characteristics for films with O/C 0.75, while defect density increases as O/C decreases. The increasing disorder most likely originates from individual aerosol gel flake edges.

### 3.3 UV–Vis Spectroscopy

The UV–vis absorption peak at ∼270 nm can be attributed to the π–π* transition of aromatic systems, such as those present in graphene and its derivatives like graphene oxide and reduced graphene oxide.^31^ This transition occurs when an electron is excited from the highest occupied π molecular orbital to the lowest unoccupied π* molecular orbital. The exact position of the peak can depend on factors such as the size and shape of the aromatic system and the presence of functional groups such as epoxides or OH groups.

The π–π* transition of graphene is an electronic transition that could occur when an electron is excited from a bonding π orbital to an antibonding π* orbital. In other words, it involves the excitation of an electron from the valence band to the conduction band. This transition is associated with an absorption peaking in the ultraviolet region, typically around 260–270 nm, and trailing across the visible range, thus responsible for the characteristic black color of graphene.^32^

### 3.4 Ultrafast Transient Absorption Spectroscopy

The transient changes in absorption/transmission (Δ*T*/*T*) of all the samples following excitation with a 400 nm laser pulse are shown in Figure 5 for both the visible (420–690 nm) and near-IR spectral regions (850–1600 nm). Immediately after excitation, all the films display positive Δ*T*/*T* signal, spanning the entire spectral range of the transient experiments. This signal is assigned to photobleaching of the broad ground-state absorption band due to depletion of the available valence to conduction band electronic transitions. For all films, the transient maximum positive signal in the visible region (500–650 nm) is reached within a pump–probe delay (*t*_max_) of 65 fs, while *t*_max_ for probe wavelengths below 500 nm increases to ∼80 fs. Conversely, in the near-IR region, *t*_max_ exhibits stronger probe wavelength dependence in all samples: *t*_max_ increases as the probe wavelength increases, apart from AG 0.75 for which the increase in *t*_max_ is negligible. The observed variation is as large as 180 fs for AG 0.5 (see the SI for graphs of *t*_max_ vs wavelength). The delay in reaching the maximum Δ*T*/*T* signal for longer wavelengths correlates with an initial negative Δ*T*/*T* signal in the region >1400 nm for AG 0.3, 0.4 and 0.5, except for AG 0.75, which is characterized by an absence of negative Δ*T*/*T* signal and a *t*_max_ of about 80 fs. These early signals however fall within our instrument response function (IRF, of ∼150 fs), which coincide with the expected coherent artifact signal (from quartz) to the overall Δ*T*/*T* signal across the visible region. Indeed, the coherent artifact signal from quartz is negative at time zero but its amplitude is not large enough to produce the observed Δ*T*/*T* response. The negative Δ*T*/*T* is a signature of the samples. An example of kinetic a trace at 1550 nm for AG 0.5, overlaid with the coherent artifact response at 1550 nm is given in the SI. In addition to this effect, the delay in reaching maximum Δ*T*/*T* signal in the near-IR region, relative to the visible region, may reflect the time required for carriers to equilibrate at energies significantly less than that of the initial photoinjected carriers (*E* = *hc*/2λ, λ = 400 nm).

**Figure 5:**
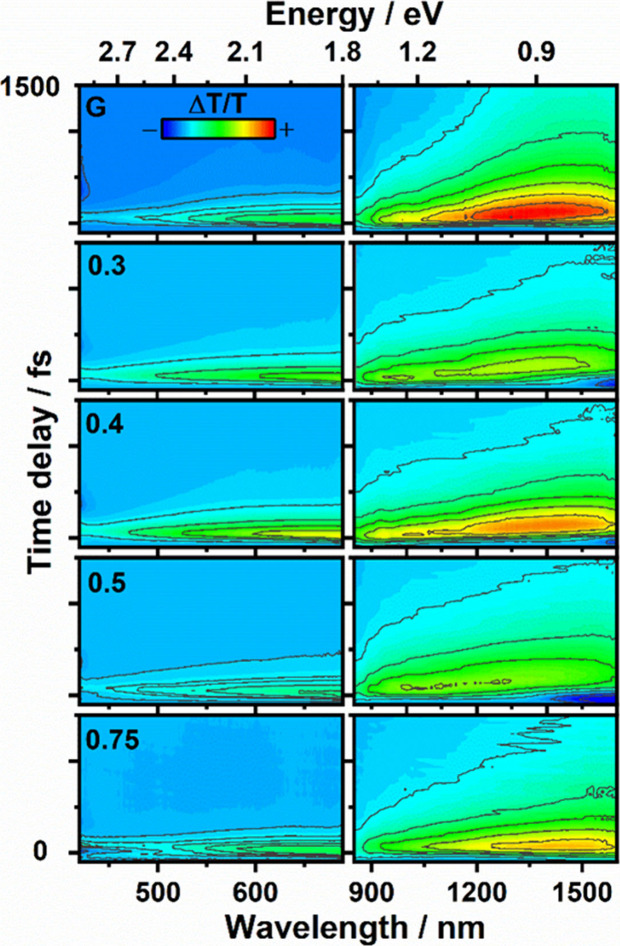
Transient changes in transmission (Δ*T*/*T*) of G and AG 0.3–0.75, following excitation at 400 nm, in the spectral regions 420–690 nm and 850–1600 nm. Pump–probe time delays span −100 to 1500 fs.

Kinetics traces at 550 and 1550 nm for the films are shown in Figure 6 and Figure 7, along with overlaid multiexponential fittings (solid curves). In the 50–1500 fs time range (Figure 6), the initial bleaching signal at 550 nm fully recovers for all samples, although the recovery is noticeably quicker for AG 0.75. Exponential fittings reveal decay times of ∼100 fs for G, AG 0.3, 0.4, 0.5, and <70 fs for AG 0.75. At 1550 nm, decay of the bleach signals occurs at slower rates (relative to λ = 550 nm) for all samples, reflecting the slower carrier–optical phonon (C–OP) scattering rates at significantly lower carrier energies.

**Figure 6:**
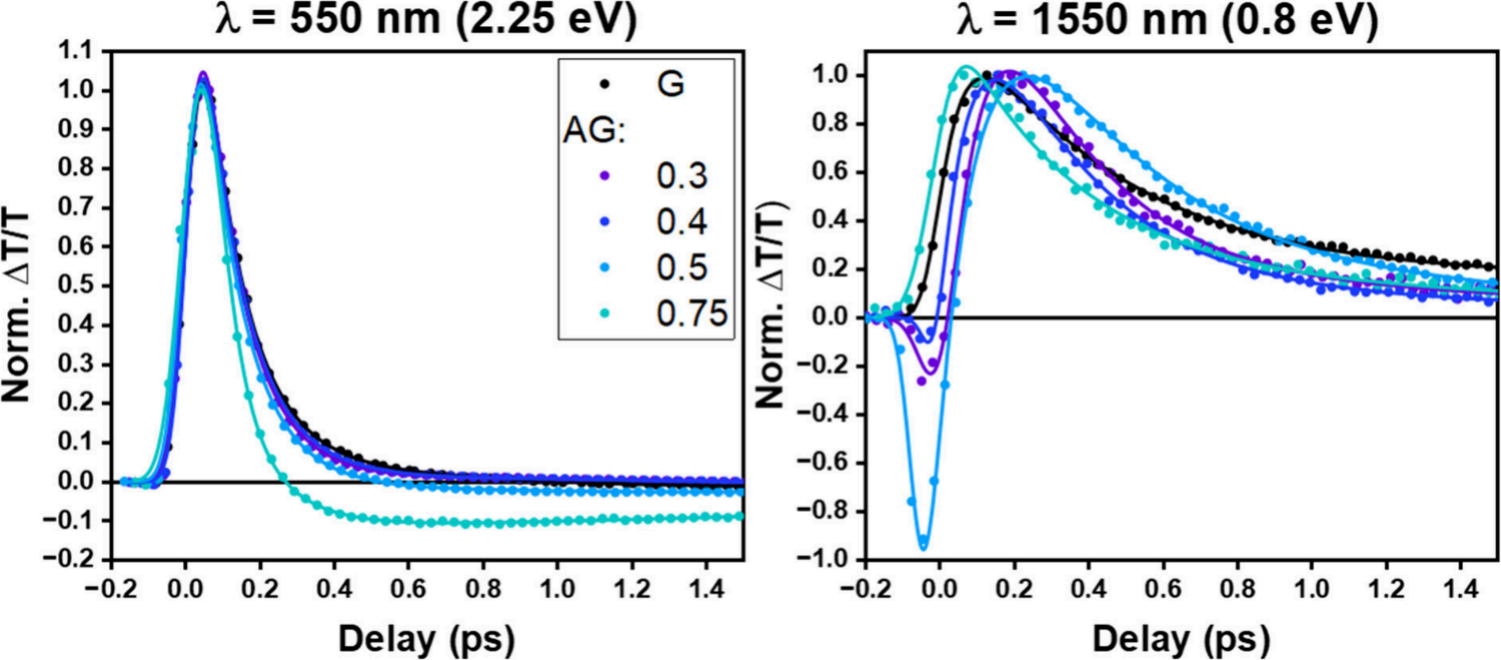
Normalized single point kinetic traces at 550 nm (left) and 1550 nm (right), for all samples, overlaid with their exponential fits.

**Figure 7:**
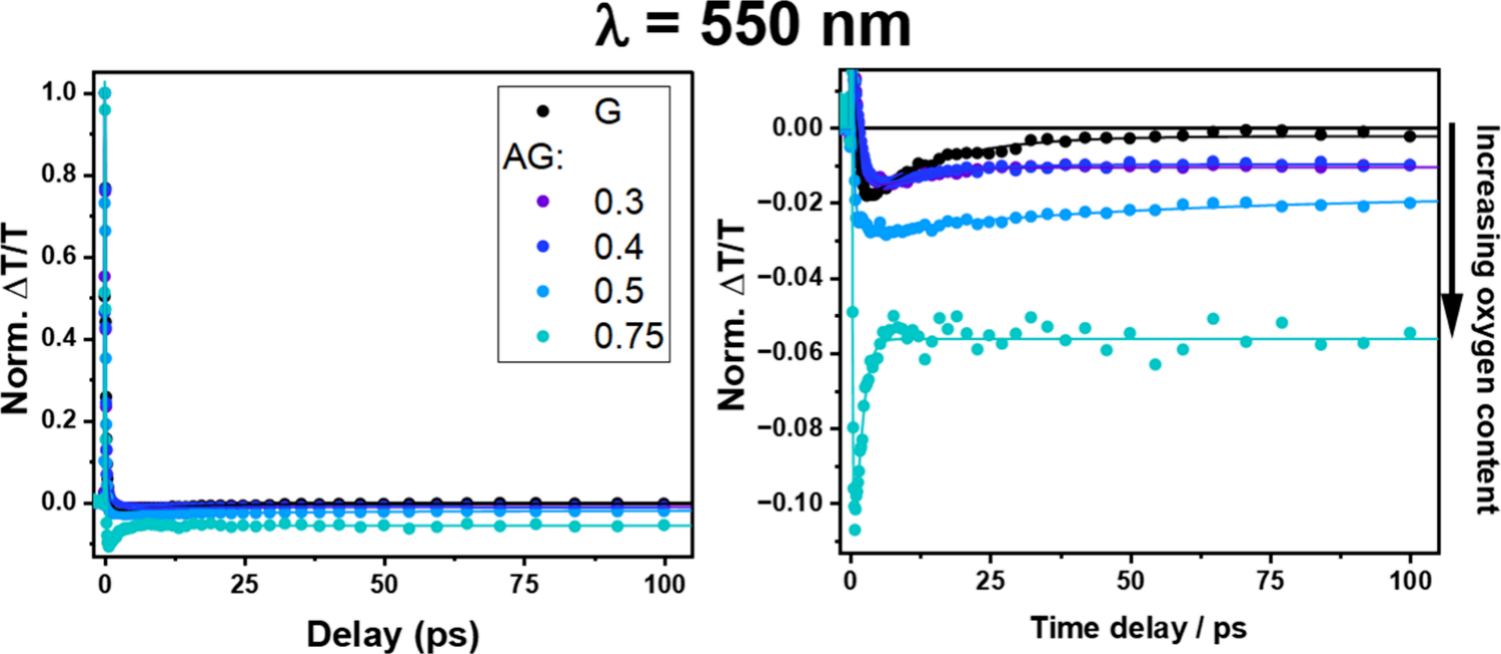
Normalized 550 nm kinetics up to pump–probe delays of 80 ps. Right panel shows the expanded photoinduced absorption (negative Δ*T*/*T*) of the same kinetics.

Decay of the initial positive Δ*T*/*T* signal exhibits a strong dependence on probe wavelength for all samples: the decay time increases as the probe wavelength increases. Plots of the C-OP scattering rates as a function of probe wavelength are given in the SI.

The long-lived transient signal is associated with absorption because of the presence of trapped electronic states. Previous work highlighted the presence of trapped states in the aerosol gel graphene films but not in pure graphene films.^33^

When looking at longer delay times (up to 100 ps, Figure 7), a clear trend in the magnitude of the negative signal is observed: G displays negligible signal at 80 ps, while for the AG films, the quasi-stationary signal increases as O/C increases from 0.3 to 0.75. Assuming that this long-living signal corresponds to trapped carriers, we are witnessing a direct correlation between an increase in the concentration of trapped carriers and O/C ratios. The exponential decay components extracted from the kinetic fits at 550 and 1550 nm are presented in Table 1 and Table 2, respectively. An alternative approach to better appreciate the correlation between the magnitude of the long-lived signal and the O/C ratio is to compare the pre-exponential factors from these kinetic fits. Note that the nondecaying signal is modeled as an infinite lifetime, τ_∞_, whose amplitude is denoted *A*_∞_. To remove the possible fluctuation in excitation energies, the correlation is illustrated by computing the ratio of *A*_∞_ over the amplitude of the initial decay lifetime (τ_1_), *A*_1_. This ratio thus represents the relative magnitude of the long-lived signal (*A*_∞_) when effectively normalized to the initial’s sample excitation (*A*_1_). Figure 8 depicts the variation in *A*_∞_/*A*_1_ as a function of O/C, with O/C of the pure graphene film being defined as 0.

**Table 1:** Lifetimes of All Samples at 1550 nm (UV–Vis)

kinetic lifetimes at 550 nm								
	subps/ps		growth neg signal/ps		recovery/ps		recovery	
sample	τ1 (ps)	τ1 error	τ2 (ps)	τ2 error	τ3 (ps)	τ3 error	τ4 (ps)	τ4 error
**G**	0.114	0.003	0.643	0.092	13.530	3.509	∞	0
**0.3**	0.115	0.002	1.643	0.755	6.651	7.579	∞	0
**0.4**	0.114	0.002	1.421	0.381	10.104	8.671	∞	0
**0.5**	0.120	0.002	0.690	0.258	54.206	26.386	∞	0
**0.75**	0.086	0.008	0.699	n/a	1.006	n/a	∞	0

**Table 2:** Lifetimes of All Samples at 1550 nm (NIR); Infinite Lifetimes Indicate Lifetimes That Are Not Decaying within Our ∼100 ps Time Window

kinetic lifetimes at 1550 nm						
sample	τ1 (ps)	τ1 error	τ2 (ps)	τ2 error	τ3 (ps)	τ3 error
**G**	0.058	0.007	0.436	0.013	∞	0
**0.3**	0.085	0.009	0.255	0.022	1.597	0.192
**0.4**	0.079	0.005	0.290	0.017	1.805	0.298
**0.5**	0.121	0.010	0.290	0.047	1.085	0.128
**0.75**	0.339	0.015	2.138	0.262	n/a	

**Figure 8:**
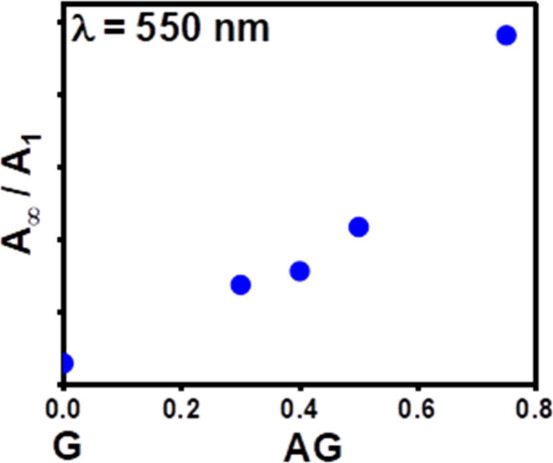
Ratio of the preexponential factors, *A*_∞_ and *A*_1_ as a function of O/C of the aerosol gel films. O/C of the pure graphene film is defined as 0.

Both Figures 7 and 8 illustrate the clear relation that exists between higher oxygen content and the increased concentration of long-lived trapped electronic states. We remind that, as exemplified in Figures 3 and 4, the increased oxygen content is intrinsically accompanied by morphological changes. Although our previous study demonstrates that the observed spectral changes mainly result from the presence of oxygen rather than from morphological changes,^1^ we cannot dismiss the possibility that part of the effect here monitored results from these structural changes. In other words, although the presence of oxygen is expected to be the dominant tuning factor, the associated changes in the morphology can play a secondary role. Overall, these results underscore the critical role of oxygen in tuning the electronic properties of graphene aerosol–gel films, opening pathways for designing materials with tailored charge-carrier relaxation for diverse technological applications.

### 3.5 Theoretical Modeling

To investigate the possible nature of the trap states, density functional theory calculations of oxygen-containing graphene were carried out. The exact chemical nature of trap states in oxygen-containing graphene is as yet unclear and is a subject of debate in the literature; such states are generally attributed to defect states and oxygen functional groups.^34^ Here we investigate substitutional oxygen as a possible origin of trap states, since high-resolution scanning transmission electron microscopy (STEM) studies demonstrated the presence of several bonding configurations of oxygen atoms in graphene,^35^ and recent theoretical calculations showed that substitutional oxygen affects the electronic properties of graphene, in particular its electrical conductivity.^36^ To investigate the possible role of substitutional oxygen in forming trap electronic states, two configurations were studied in this work: graphene containing a triple-coordinated oxygen atom (Figure 9, left) and a pair of two-coordinated oxygen atoms (Figure 9, right). Both structures were reported in the STEM study,^35^ although the latter was more frequently observed.

**Figure 9:**
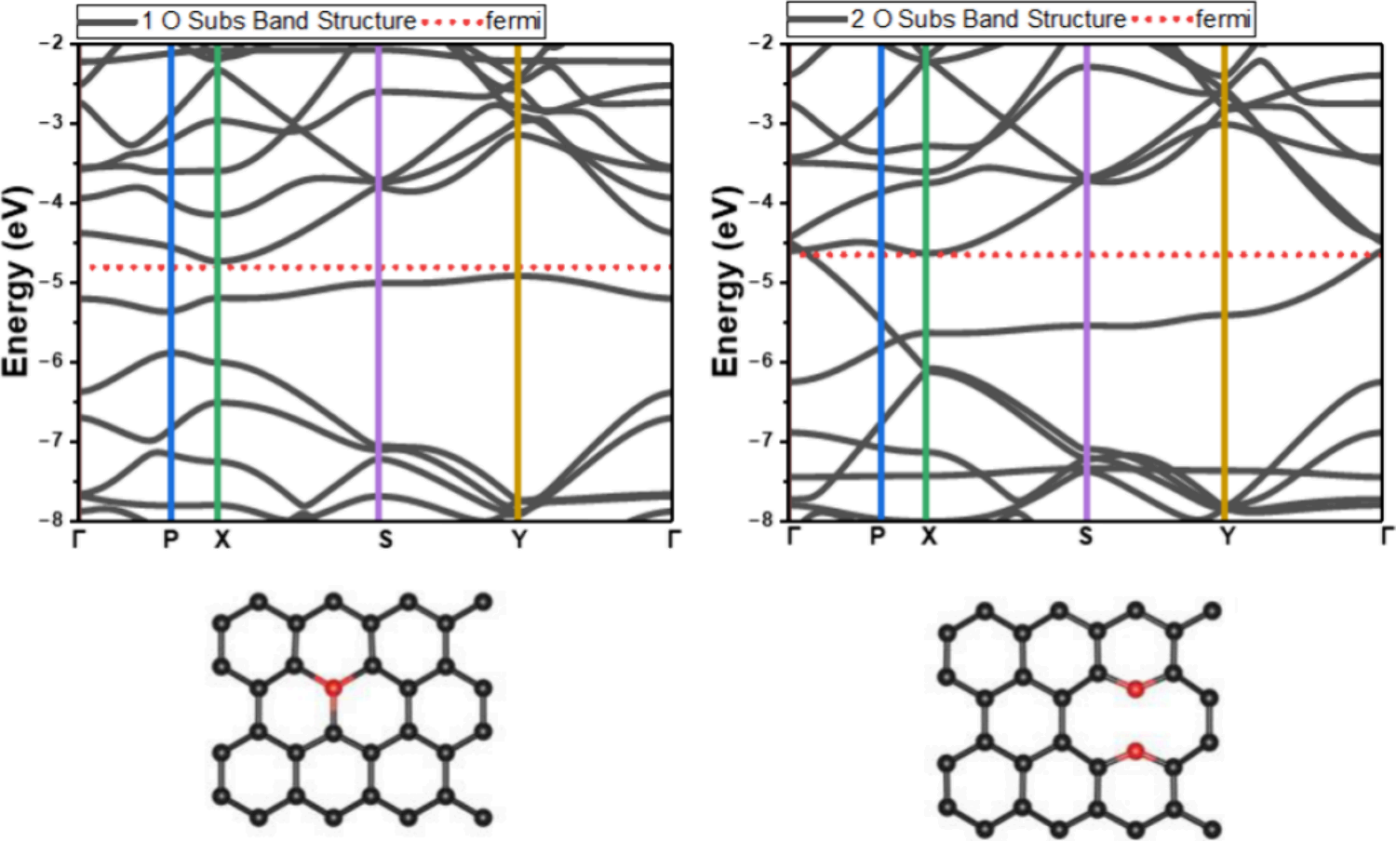
Calculated band structures of graphene containing (left) a triple-coordinated substitutional oxygen atom and (right) a pair of two-coordinated substitutional oxygen atoms. Energies are reported relative to the vacuum level, which is set as zero. The horizontal dotted line shows the position of the Fermi level.

The band structure plots shown in Figure 9 show that the presence of a triple-coordinated oxygen atom gives rise to a flat band just below the Fermi level, which is likely to act as electron trap state. In contrast, the pair of two-coordinated oxygens gives rise to a flat band above the Fermi level in the Γ–X direction, which is likely to act as a hole trap state. Therefore, various arrangements of substitutional oxygen in graphene may be responsible for charge trap states inferred from ultrafast transient absorption spectroscopy results.

## 4 Conclusion

In summary, our investigation into graphene films made of aerosol–gel printable inks with varying oxygen contents reveals a direct correlation among oxygen levels, material morphology, and ultrafast carrier dynamics. UV–vis spectroscopy showed broadened absorption with increasing oxygen content, increasing defect densities, increasing particle size, and varying morphology in graphene aerosol gel inks. More specifically, our pump–probe studies clearly established that higher oxygen incorporation directly correlates with an augmented population of long-lived trapped electronic states. On the basis of the results of our DFT calculations, these trapped states may be attributed to the presence of substitutional oxygen. This work provides fundamental insights into the mechanisms governing carrier relaxation in these tunable graphene systems, paving the way for graphene-based functional materials. Understanding and control of carrier relaxation pathways of graphene will enable its use in a wide variety of applications, such as sensing and biosensing, bioimaging, and lighting and display techniques.

## Supplementary Material



## Notes

Correlation between fs-decay component and probe wavelength from 420 to 1500 nm,^2^ comparison between coherent artifact in bare quartz and in graphene aerosol gel samples, and^3^ correlation between decay components and the sample’s oxygen content (PDF)
