# Supplementary material for: Impact of Morphology and Composition of Graphene Aerosol–Gel Particles in Thin Films on Ultrafast Carrier Dynamics Studied via Transient Absorption Spectroscopy
Source: J Phys Chem C Nanomater Interfaces. 2026 Jun 5;130(24):8417–25. doi: 10.1021/acs.jpcc.6c02864 (PMC13288620; doi:10.1021/acs.jpcc.6c02864)
Supplement: Supplementary file 1 [file jp6c02864_si_001.pdf]

Supporting Information File

Impact of Morphology and Composition of Graphene Aerosol-Gel Particles in Thin Films on Ultrafast Carrier Dynamics Studied via Transient Absorption Spectroscopy

Alexander J. Auty, [1,+] *Negar Mansouriboroujeni*[1,+], *Thiba Nagaraja,*[2,+], *Dimitri Chekulaev,* [1] *Natalia Martsinovich,*[1] *Suprem R. Das,*[2,3,*], *Adrien A. P. Chauvet* [1,*]

[1] School of Mathematics and Physical Sciences, University of Sheffield, Sheffield, S3 7HF, UK

[2] Department of Industrial and Manufacturing Systems Engineering, Kansas State University, Manhattan, Kansas, 66506, USA

[3] Department of Electrical and Computer Engineering, Kansas State University, Manhattan, Kansas, 66506, USA

This Supplementary Information file contains:

- A visual depiction of the correlation between the fs-decay compenent and probe wavelength from 420 nm to 1500 nm (Figure S 1).
- A comparison between the coherent artifact in bare quartz and in the graphene aerosol gel samples (Figure S 2).
- A visual depiction of the correlation between the various decay compenent and the sample's oxygen content (Figure S 3).

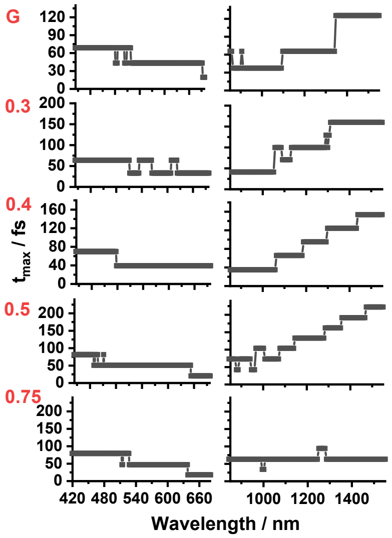

Figure S 1: Correlation between fs-decay time and probe wavelength, illustrative of the increase of carrier–optical phonon scattering rate as a function of probe wavelength.

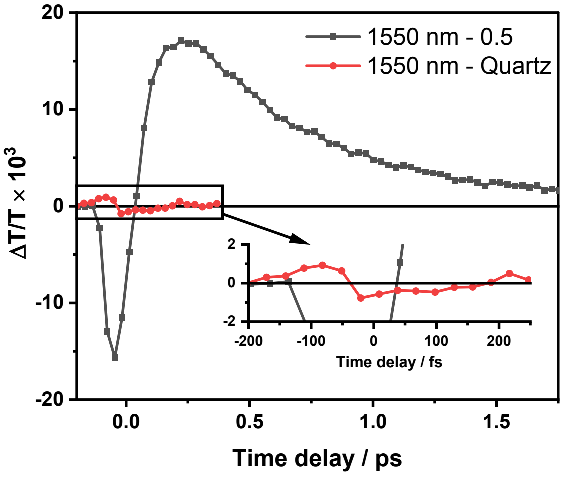

Figure S 2: Early ΔT/T kinetic signals of AG 0.5 sample at 1550 nm overlaid with the coherent artifact response from the Quartz cuvette at the same wavelength.

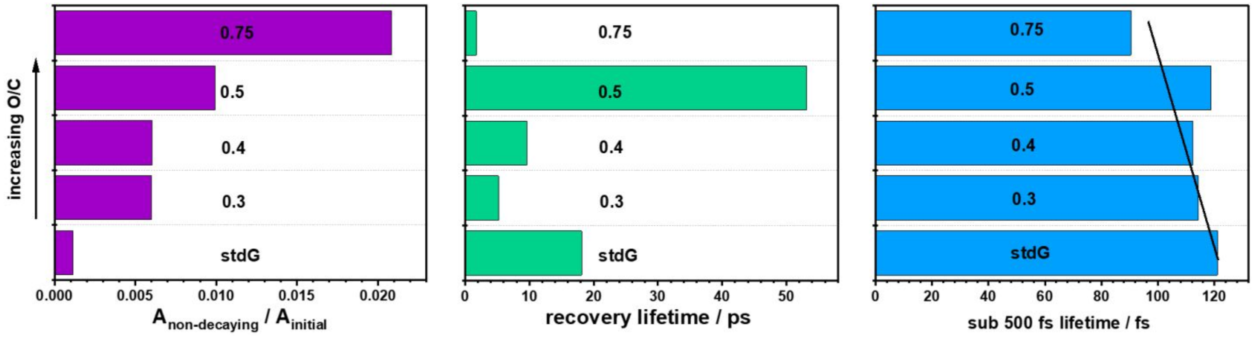

Figure S 3: Oxygen content versus amplitude ratio (left) and sub-ps lifetime (right) showing clear trends: As the oxygen content increases, the long-living signal amplitude increases and the trapping lifetime decreases. There is however no clear trend in ground state recovery lifetime (middle).
